# Atomic-Scale Modulation of Lithium Metal Electrode Interfaces by Monolayer Graphene: A Molecular Dynamics Study

**DOI:** 10.3390/ma18214925

**Published:** 2025-10-28

**Authors:** Haoyu Yang, Runze Chen, Shouhang Fu, Shunxiang Mo, Yulin Chen, Jianfang Cao

**Affiliations:** 1 School of Chemical Engineering, Ocean and Life Sciences, Dalian University of Technology, Panjin 124221, China; 2533181638@mail.dlut.edu.cn (H.Y.); xingyun@mail.dlut.edu.cn (S.M.); 2 Faculty of Medicine, Dalian University of Technology, Dalian 116024, China; runzec0729@163.com (R.C.); fushouhang@mail.dlut.edu.cn (S.F.); 3 School of Physical Education, Wuhan University of Technology, Wuhan 430062, China; wutcyl@163.com

**Keywords:** graphene, lithium anode, calendering, molecular dynamics

## Abstract

Graphene, owing to its exceptional mechanical properties and interfacial modulation capability, is considered an ideal material for enhancing the interfacial strength and damage resistance during the fabrication of ultra-thin lithium foils. Although previous studies have demonstrated the reinforcing effects of graphene on lithium metal interfaces, most analyses have been restricted to single-temperature or idealized substrate conditions, lacking systematic investigations under practical, multi-temperature environments. Consequently, the influence of graphene coatings on lithium-ion conductivity and mechanical stability under real thermal conditions remains unclear. To address this gap, we employ LAMMPS-based molecular dynamics simulations to construct atomic-scale models of pristine lithium and graphene-coated lithium (C/Li) interfaces at three representative temperatures. Through comprehensive analyses of dislocation evolution, root-mean-square displacement, frictional response, and lithium-ion diffusion, we find that graphene coatings synergistically alleviate interfacial stress, suppress crack initiation, reduce friction, and enhance ionic conductivity, with these effects being particularly pronounced at elevated temperatures. These findings reveal the coupled mechanical and electrochemical regulation imparted by graphene, providing a theoretical basis for optimizing the structure of next-generation high-performance lithium metal anodes and laying the foundation for advanced interfacial engineering in battery technologies.

## 1. Introduction

Calendering is a pivotal process in the industrial fabrication of high-energy-density lithium metal batteries, enabling the production of ultra-thin lithium foils (≤20 um) required for high-capacity electrodes [1,2,3,4]. However, previous studies have shown that micro- and nanoscale asperities on the roll surface can easily damage the lithium interface during calendering, leading to crack formation, surface amorphization, and ultimately degradation of the electrode’s cycling stability and safety [5,6,7].

To address interface degradation during calendering, a variety of interfacial engineering strategies have been developed to improve the mechanical integrity and damage tolerance of lithium metal under compressive stress [8,9,10]. Among various candidate materials, graphene has emerged as a particularly promising interfacial reinforcement owing to its unique mechanical properties and capacity for interfacial modulation. Previous studies have demonstrated that graphene exhibits pronounced interfacial enhancement effects on lithium metal anodes under specific thermal conditions, primarily attributable to its exceptional mechanical strength and intrinsic lubricity. For example, Yu et al. [11] reported that graphene-coated lithium foils prepared under isothermal conditions exhibited significantly improved cycling stability in pouch cells. Similarly, Lv et al. [12] employed a three-dimensional nitrogen-doped graphene aerogel as a host for lithium metal anodes at a constant temperature, resulting in uniform and dendrite-free lithium deposition. Furthermore, Zhou et al. [13] utilized molecular dynamics simulations to reveal that, under single-temperature conditions, monolayer graphene coatings on copper or silicon substrates substantially reduced interfacial friction and enhanced interfacial mechanical stability. While existing studies have confirmed graphene’s ability to reinforce lithium–metal interfaces, most rely on single-temperature simulations or idealized substrates and thus fail to clarify how graphene coatings affect both lithium-ion conductivity and mechanical stability across realistic thermal conditions. This limitation hampers rational interface and electrode design for next-generation lithium-metal batteries.

To address this gap, atomic-scale models of pristine lithium and graphene-coated lithium (C/Li) interfaces were constructed. The selected temperatures (293 K, 300 K, and 373 K) are within or near the typical range used for pure lithium calendering (298–343 K). The highest temperature, 373 K, reflects conditions occasionally required for enhanced plasticity during specialized processing. These simulations systematically evaluated interfacial responses under nanoindentation and sliding, including dislocation evolution, frictional behavior, and lithium-ion diffusion.

Distinct from previous studies confined to a single temperature, this work systematically demonstrates the pronounced temperature dependence of mechanical reinforcement and ionic conductivity at graphene-coated lithium interfaces. At elevated temperatures, where lithium is more susceptible to plastic deformation, graphene coatings significantly promote stress relaxation and enhance lithium-ion mobility. These findings reveal the temperature-coupled relationship between interfacial stability and ion conduction at the graphene–lithium interface, providing critical mechanistic insights for interface design of lithium metal anodes under practical processing conditions. Moreover, these mechanistic insights lay a theoretical foundation for integrating graphene with other two-dimensional materials in multilayer or hybrid interfacial coatings, thereby advancing the optimization of lithium metal anodes.

## 2. Molecular Dynamics Simulation Methodology

**Figure 1:**
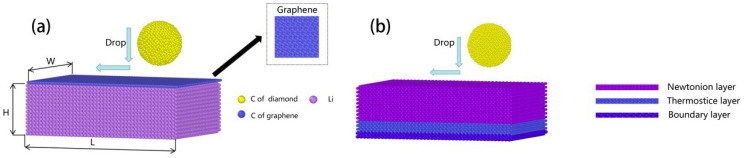
Model construction diagram: (**a**) Model of graphene-monolayer-covered Li-metal (Li/C) and its atomic list; (Li/C); (**b**) Model of Li-metal and functionally partitioned layers.

In order to investigate the mechanical response and transport properties of graphene-covered lithium-metal anode interfaces, molecular dynamics simulations were performed in this paper using LAMMPS (2021) [14] software. The size of the simulated system is 151.62 Å × 101.08 Å × 48 Å, which contains a lithium metal matrix, a graphene-covered layer and a diamond probe. The lithium metal matrix has a body-centered cubic (BCC) structure with a lattice constant of 3.44 Å. The graphene layer is a single hexagonal lattice with a lattice constant of 2.4768 Å, which is placed on the surface of the lithium metal to construct a composite interface. In this study, two simulated systems were constructed: Figure 1a Lithium-metal interface (Li/C) with surface covered with a single layer of graphene; Figure 1b unmodified pure lithium-metal interface (Li). Among them, the C/Li system was simulated at 293 K, 300 K and 373 K, respectively, to investigate the effect of temperature on the properties of the LI/C interface.

Classical physical potential functions are adopted within the materials, including the AIREBO potential function for C-C interatomic interactions within graphene and diamond probes [15]; and the widely validated MEAN potential function for Li-Li interatomic interactions [16]. The Lennard-Jones (LJ) potential function is used for non-bonding interactions between the diamond probe and the matrix material [17]. The LJ potential parameters are ε = 0.0022217 ev and σ = 2.8072 Å for C-C and ε = 0.00286 ev and σ = 3.47 Å for Li-Li, where ε represents the depth of the potential and σ represents the zero crossing point of the potential [18].

The overall simulation protocol was designed in two sequential phases: a nanoindentation stage followed by a nano-friction stage. During the nanoindentation stage, a diamond probe was driven into the substrate along the *Z*-axis at a constant penetration rate of 0.5 Å/ps, while the temporal evolution of indentation depth and the corresponding variations in normal load were continuously monitored and recorded. After reaching the predetermined indentation depth, the simulation transitioned into the nano-friction stage, wherein the same probe was laterally displaced along the *X*-axis over a sliding distance of 150 Å at an equivalent velocity. This configuration was implemented to realistically replicate the frictional response and contact behavior that occurs during practical roll-pressing operations at the nanoscale.

The visualization of the atomistic simulation outcomes was primarily conducted using the OVITO package, which enables detailed structural analysis at the nanoscale. To elucidate the impact of distinct interfacial architectures on the interfacial properties of lithium metal, this study systematically quantifies the localized plastic deformation processes and evaluates their regulatory effect on the overall structural stability of the material. This quantification was achieved by deriving both the spatial distribution of dislocation networks and the statistical characterization of dislocation lengths.

With respect to interfacial frictional characteristics, the friction coefficient (μ) was determined from the ratio of the simulation-derived frictional force (F_f_) to the corresponding normal force (F_n_). This parameter serves as a key quantitative descriptor for assessing interfacial lubrication efficiency and shear resistance across different material systems [19].

(1) $$\mu =\frac{F_{f}}{F_{n}}$$

In the analysis of ion transport behavior, the radial distribution function (RDF) was employed as a principal tool to characterize the degree of atomic ordering within the interfacial region, thereby elucidating the critical contribution of the graphene overlayer to enhanced structural regularity and improved thermal stability. In parallel, mean-square displacements (MSDs) were systematically computed, and from these trajectories the diffusion coefficient (D) was rigorously derived, providing a quantitative descriptor of ion mobility and transport efficiency in the examined systems [20].

(2) $$D=\frac{\mathrm{MSD}}{6t}$$

The electrical conductivity (σ) is further estimated by Nernst-Einstein equation [21].

(3) $$\sigma =\frac{nq^{2}D}{K_{B}\;T}$$

where K_B_ is the Boltzmann constant with a magnitude of 1.381 × 10^−23^ J/K; q denotes the carrier charge, with a magnitude of 1.60 × 10^−19^ C; and the remaining parameters are defined in Table 1.

## 3. Results and Analysis

### 3.1. Dislocation

During the calendering process, the nucleation and subsequent evolution of dislocations within the metallic matrix exert a decisive influence on the overall service life, mechanical robustness, and electrochemical performance of lithium metal batteries. Thus, a comprehensive and systematic investigation of dislocation dynamics under sustained mechanical deformation is indispensable, as highlighted by prior studies [22]. Within the framework of graphene-reinforced lithium metal systems, dislocation behavior is predominantly dictated by three principal types: 1/6< 112 > stair-rod dislocations, 1/3< 110 > Hirth dislocations, and other miscellaneous dislocations. The present study is dedicated to meticulously tracking the progression and transformation pathways of these representative dislocation types under externally applied loading. Such detailed analysis enables the elucidation of the underlying mechanisms governing defect evolution and structural adaptation at the atomic scale, ultimately providing critical insights for the rational design and optimization of advanced lithium metal electrode materials with enhanced durability, mechanical reliability, and electrochemical stability.

**Figure 2:**
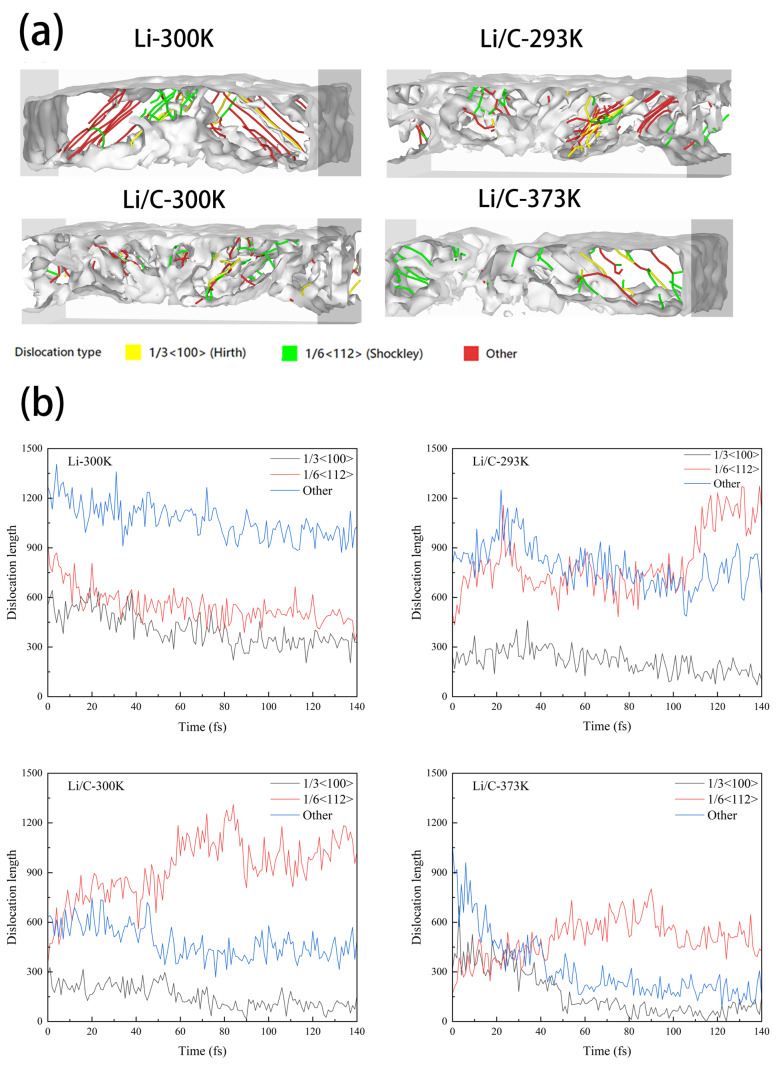
Dislocation map: (**a**) Dislocation distribution; (**b**) Dislocation length.

Figure 2 presents a comprehensive three-dimensional visualization of dislocation distributions and the corresponding structural transformations in both pristine lithium and graphene-coated lithium matrices, as observed through molecular dynamics simulations. At a representative temperature of 300 K, during the progression of nanoindentation and nanofriction, standard dislocations in pure lithium (including the 1/3< 110 > Hirth and 1/6< 112 > Shockley dislocations) transform into more complex, irregular, and energetically unstable dislocation configurations. This transformation leads to the emergence of highly localized, heterogeneous, and disordered slip pathways, which undermine the mechanical coherence and structural integrity of the material. In contrast, the graphene-coated Li/C system exhibits markedly different behavior. Specifically, the number of 1/6< 112 > Shockley dislocations increase steadily over time, while 1/3< 110 > Hirth dislocations gradually decrease. Notably, the standard dislocations in this composite system rarely evolve into irregular, non-standard forms. This observation indicates that the graphene interface serves as an effective barrier, suppressing the transformation of regular dislocations into energetically unfavorable complex configurations. As such, the graphene layer significantly enhances interfacial mechanical stability by preserving the structural order of dislocation networks.

As temperature increases, thermal vibrations in the Li/C system intensify, leading to a pronounced reduction in overall dislocation density. This effect is chiefly ascribed to enhanced atomic mobility at elevated temperatures, which promotes more efficient dislocation annihilation and rearrangement. As a result, defect accumulation is mitigated and the structural order of the matrix is maintained. Concurrently, the spatial distribution of dislocations becomes increasingly diffuse, and the fraction of irregular dislocations further diminishes. These findings suggest that elevated thermal conditions contribute to the suppression of dislocation nucleation and promote the persistence of energetically favorable, regular dislocation structures. This effect is reflected in the achievement of lower dislocation density while maintaining the overall structural integrity of the matrix. Such a result is highly significant for improving and strengthening the overall stability of the matrix material, as well as for increasing the reliability and dependability of interface engineering applications [23].

### 3.2. Frictional Forces

**Figure 3:**
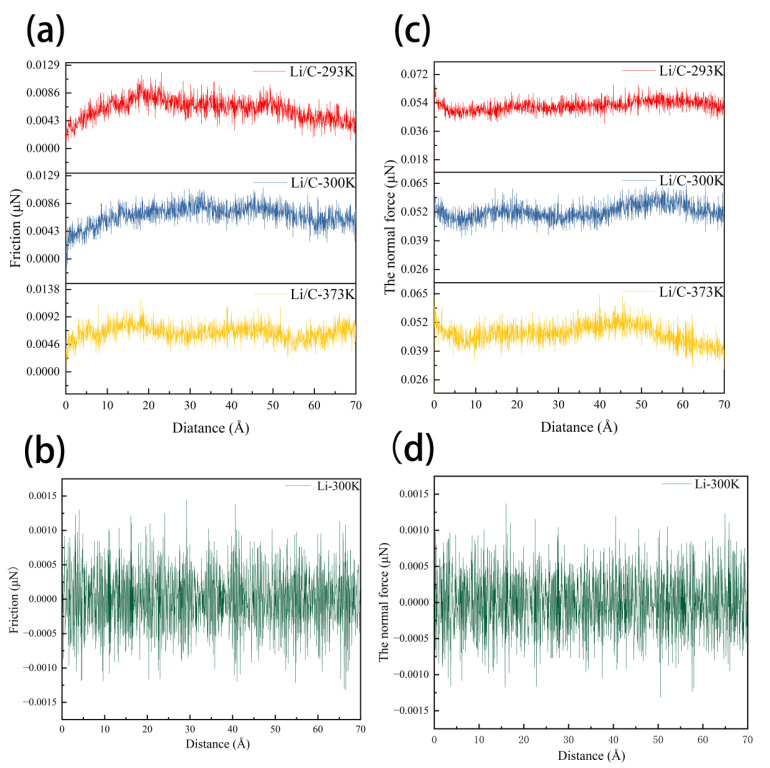
Friction Performance Chart.: (**a**) Normal force vs. distance curve for Li/C; (**b**) friction force vs. distance curve for Li/C; (**c**) normal force vs. distance curve for Li; (**d**) friction force vs. distance curve for Li.

Interfacial friction represents a critical factor dictating both the microstructural integrity and the long-term functional durability of lithium metal foils, as it regulates not only the stability of the interfacial region but also the progressive accumulation of mechanical damage during extended service [10]. To elucidate these mechanisms, we systematically analyzed the evolution of frictional and normal forces in both pristine lithium and graphene-coated lithium (Li/C) composite systems under shear deformation, as depicted in Figure 3. In the pristine lithium system, frictional force exhibits a relatively smooth and stable trajectory, and the normal force undergoes only minor fluctuations. This behavior indicates that the surface readily undergoes plastic deformation in response to shear loading, which allows the interface to rapidly adapt to changing external forces and results in low mechanical hysteresis (Figure 3b). Such characteristics are indicative of limited energy storage capacity and a tendency toward irreversible structural changes under repeated dynamic loads.

By contrast, the Li/C composite system demonstrates a markedly different mechanical response. Both frictional and normal forces exhibit pronounced and periodic fluctuations, with a one-to-one correspondence between peaks and troughs (Figure 3a). This periodicity suggests that the graphene-coated interface is significantly more sensitive and responsive to dynamic loading, reflecting a fundamental shift in the way the interface accommodates external stress. The origin of this phenomenon can be primarily attributed to the enhanced interfacial compliance and adaptive stress distribution enabled by the graphene layer, which allows the interface to store and dissipate mechanical energy in a more controlled manner. Li et al. [24] used molecular dynamics simulations to show that introducing graphene into composite interfaces can regulate local deformation and facilitate more efficient stress transfer, thereby improving both mechanical and tribological performance. Liang et al. [8] further demonstrated that graphene-based surface engineering techniques enhance the structural stability of lithium metal anodes by optimizing interfacial architecture and energy dissipation pathways, especially under practical operating conditions. Moreover, Galiakhmetova et al. [25] reported that graphene significantly modulates dislocation dynamics and stress distribution at elevated temperatures, further supporting its role in interfacial viscoelasticity and adaptive response. These results are highly consistent with the conclusions of previous studies and provide direct evidence for the role of graphene in regulating the periodic interfacial response of lithium metal systems.

A comparative analysis of friction coefficients, as summarized in Table 2, further supports these conclusions. At 300 K, the friction coefficient of the Li/C interface is reduced by nearly 90% relative to pristine lithium, demonstrating that the graphene coating is highly effective in suppressing plastic slip and mitigating the accumulation of microscopic damage in the contact zone. This dramatic reduction in interfacial resistance results in much smoother sliding behavior and a lower risk of interface failure during calendering. Furthermore, as the operational temperature increases, the friction coefficient of the Li/C system exhibits only a negligible upward trend, indicating that the lubrication and adaptive properties of the graphene-modified interface are preserved even under elevated temperatures.

In summary, the introduction of a graphene interfacial layer provides stable and precise control over both frictional and normal mechanical loads by enhancing interfacial smoothness and elastic compliance. This approach suppresses shear stress concentration and mitigates the risks associated with uncontrolled interfacial slippage, which can arise from surface asperities or microstructural defects. Collectively, these findings highlight the exceptional potential of graphene as an advanced interface engineering material for improving the mechanical performance and operational reliability of lithium metal foils during industrial calendering.

### 3.3. Radial Distribution Function

The radial distribution function (RDF) reflects the spatial distribution and degree of ordering of atoms in the interfacial region, serving as a key parameter for characterizing the stability and ordering of a material’s microstructure [26,27]. To this end, the RDF curves of different systems at various temperatures were derived in this study to quantitatively evaluate the ordering and structural stability of interfacial atoms.

**Figure 4:**
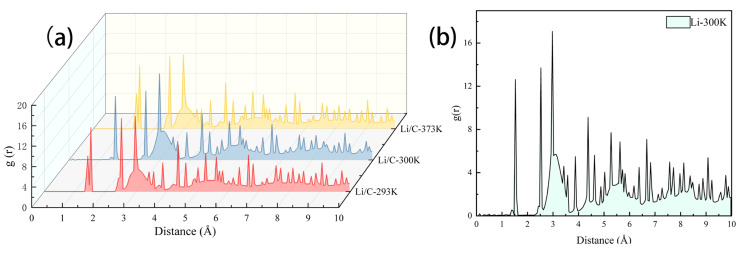
Plot of radial distribution function-(**a**) Li/C system; (**b**) Pure Li system.

As shown in Figure 4b, at 300 K, the RDF curve for pure lithium displays a broad and low-amplitude main peak, indicating significant thermal perturbation and a high degree of local disorder. By contrast, the Li/C system exhibits a sharper main peak and more pronounced secondary peaks, indicating enhanced structural compactness at the graphene-modified interface [28].

Furthermore, as shown in Figure 4a, the RDF of the Li/C system changes only minimally as the temperature increases from 293 K to 373 K, suggesting that the presence of graphene helps to maintain interfacial structural order under thermal excitation. These results provide atomic-scale evidence that graphene coatings can slow the degradation of interfacial structure under elevated temperatures.

### 3.4. Mean Square Displacementa

**Figure 5:**
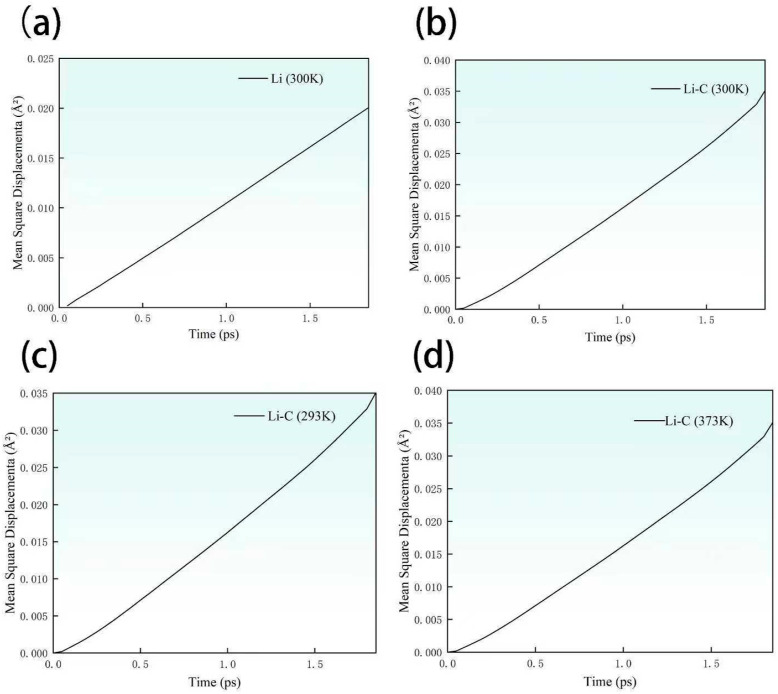
Plot of mean square displacement (**a**) Li-300 K; (**b**) Li/C-300 K; (**c**) Li/C-293 K; (**d**) Li/C-373 K.

To evaluate the regulatory influence of distinct interfacial structures on the migration capacity of charge carriers, this section presents a comprehensive analysis of the mean square displacement (MSD) for each simulated system. Based on the MSD data, the corresponding diffusion coefficients and electrical conductivities are further derived, as graphically depicted in Figure 5 and quantitatively summarized in Table 1 [29]. At 300 K, the Li/C system exhibits a significantly higher MSD value compared to the pristine Li system, indicating that the graphene overlayer markedly facilitates interfacial ion mobility. This enhancement underscores the critical role of graphene in promoting dynamic ion transport across the interface [30].

As reported in Table 1, the diffusion coefficient of the Li/C system at 300 K reaches 2.77 × 10^−11^ m^2^/s, which is approximately 1.54 times greater than that of pure lithium (1.80 × 10^−11^ m^2^/s). Simultaneously, the electrical conductivity of the Li/C interface increases by more than 80%, reflecting graphene’s ability to significantly improve both ionic diffusivity and electronic transport at the interface. These results collectively demonstrate that graphene not only contributes to enhanced structural integrity at the atomic scale but also plays a pivotal role in augmenting charge transport efficiency across the interface. This improvement can be attributed to graphene’s intrinsically layered configuration, which forms a continuous, low-resistance channel for electron conduction. Additionally, the increased atomic ordering at the modified interface promotes localized ion migration, resulting in simultaneous enhancement of both electronic and ionic transport performance [31].

Upon increasing temperature, the mean-square displacement (MSD) of the Li/C system demonstrates a progressive yet distinct decline, which is paralleled by marked decreases in both the diffusion coefficient and electrical conductivity. This response reveals that heightened thermal agitation substantially impedes long-range ion transport across the graphene-modified interface, resulting in diminished charge carrier mobility. Notably, despite exposure to pronounced thermal fluctuations up to 373 K, the Li/C system consistently maintains a relatively high level of electrical conductivity. This sustained transport performance clearly illustrates the exceptional regulatory capacity and intrinsic resilience of the graphene interfacial layer, even under severe thermal conditions. Overall, these results provide compelling evidence of the thermal robustness and functional stability afforded by graphene modification. Such insights are crucial for the rational design and development of advanced energy storage devices that demand reliable charge transport and operational stability under dynamically varying and challenging thermal environments.

## 4. Conclusions

In this study, molecular dynamics simulations were systematically employed to elucidate the atomic-scale interfacial properties of pure lithium and graphene-coated lithium (Li/C) systems during the calendering process. The investigation primarily focuses on the evolution of dislocations, interfacial frictional behavior, atomic ordering, and ion transport characteristics during both nanoindentation and nanofriction stages. The principal findings are summarized as follows. (1) The introduction of a graphene coating effectively suppresses the formation of irregular, high-energy dislocations, markedly reduces the overall dislocation density, and promotes the maintenance of well-ordered dislocation networks, particularly at elevated temperatures. (2) The graphene-modified interface exhibits enhanced viscoelastic response, enabling efficient elastic load transfer and significantly delaying the initiation of localized mechanical damage under cyclic loading. (3) The presence of graphene facilitates a higher degree of atomic ordering at the interface, thereby preserving the structural integrity of the lithium metal matrix under combined thermal and mechanical stresses. (4) The graphene-coated interface demonstrates substantially improved ionic and electronic transport properties, as evidenced by pronounced increases in both the diffusion coefficient and electrical conductivity.

Collectively, these results provide atomistic insight into the coupled mechanical and electrochemical performance of graphene-engineered interfaces. They offer guidance for the rational design of high-performance lithium metal battery interfaces and establish a theoretical basis for optimizing the mechanochemical contributions of graphene relative to lithium foil thickness during industrial calendering, as well as for developing multilayer or hybrid coatings incorporating other two-dimensional materials.

## Figures and Tables

**Table 1:** Electrochemical Properties.

Substrate Material	T (K)	*n* (Electrons/cm^3^)	MSD/t (Å^2^/ps)	D (m^2^/s)	σ (S/m)
Li	300 K	4.63 × 10^22^	1.08 × 10^−2^	1.80 × 10^−11^	5.15
Li-C	293 K	5.58 × 10^22^	1.66 × 10^−2^	2.77 × 10^−11^	9.78
Li-C	300 K	5.58 × 10^22^	1.66 × 10^−2^	2.77 × 10^−11^	9.55
Li-C	373 K	5.58 × 10^22^	1.61 × 10^−2^	2.68 × 10^−11^	7.43

**Table 2:** Friction Characteristics Diagram.

Substrate Material	Temperature	The Normal Force (μN)	Friction (μN)	Friction Coefficient
Li	300 K	5.990 × 10^−7^	6.110 × 10^−7^	1.020
Li-C	293 K	7.481 × 10^−5^	8.291 × 10^−6^	0.111
Li-C	300 K	7.465 × 10^−5^	9.138 × 10^−6^	0.122
Li-C	373 K	6.766 × 10^−5^	9.159 × 10^−6^	0.135

## Data Availability

The original contributions presented in this study are included in the article. Further inquiries can be directed to the corresponding author.
